# CoQ10 Augments Rosuvastatin Neuroprotective Effect in a Model of Global Ischemia *via* Inhibition of NF-κB/JNK3/Bax and Activation of Akt/FOXO3A/Bim Cues

**DOI:** 10.3389/fphar.2017.00735

**Published:** 2017-10-13

**Authors:** Sarah A. Abd El-Aal, Mai A. Abd El-Fattah, Hanan S. El-Abhar

**Affiliations:** ^1^Department of Pharmacology and Toxicology, October 6 University, Cairo, Egypt; ^2^Department of Pharmacology and Toxicology, Faculty of Pharmacy, Cairo University, Cairo, Egypt

**Keywords:** CA1, caspase-3, MPO, neuroprotection, oxidative stress, statins

## Abstract

Statins were reported to lower the Coenzyme Q10 (CoQ10) content upon their inhibition of HMG-CoA reductase enzyme and both are known to possess neuroprotective potentials; therefore, the aim is to assess the possible use of CoQ10 as an adds-on therapy to rosuvastatin to improve its effect using global I/R model. Rats were allocated into sham, I/R, rosuvastatin (10 mg/kg), CoQ10 (10 mg/kg) and their combination. Drugs were administered orally for 7 days before I/R. Pretreatment with rosuvastatin and/or CoQ10 inhibited the hippocampal content of malondialdehyde, nitric oxide, and boosted glutathione and superoxide dismutase. They also opposed the upregulation of gp91^phox^, and p47^phox^ subunits of NADPH oxidase. Meanwhile, both agents reduced content/expression of TNF-α, iNOS, NF-κBp65, ICAM-1, and MPO. Besides, all regimens abated cytochrome *c*, caspase-3 and Bax, but increased Bcl-2 in favor of cell survival. On the molecular level, they increased *p*-Akt and its downstream target *p*-FOXO3A, with the inhibition of the nuclear content of FOXO3A to downregulate the expression of Bim, a pro-apoptotic gene. Additionally, both treatments downregulate the JNK3/c-Jun signaling pathway. The effect of the combination regimen overrides that of either treatment alone. These effects were reflected on the alleviation of the hippocampal damage in CA1 region inflicted by I/R. Together, these findings accentuate the neuroprotective potentials of both treatments against global I/R by virtue of their rigorous multi-pronged actions, including suppression of hippocampal oxidative stress, inflammation, and apoptosis with the involvement of the Akt/FOXO3A/Bim and JNK3/c-Jun/Bax signaling pathways. The study also nominates CoQ10 as an adds-on therapy with statins.

## Introduction

Transient global cerebral ischemia is a clinical devastating predicament arising during cardiac arrest, rescindable severe hypotension, and neonatal asphyxia (Cui et al., 2016). This insult induces a selective and delayed neuronal death (DND) of hippocampal cornu ammonis 1 (CA1) neurons within 3–7 days after its occurrence (Colbourne et al., 1999).

The mechanisms underlying ischemia reperfusion (I/R)-induced neuronal death implicate a complex interplay of myriad pathways, including excitotoxicity, formation of reactive oxygen species (ROS), release of inflammatory mediators, calcium overload, and upregulation of apoptotic genes (Wang et al., 2016). Oxidative stress (OS) has been involved in the progression of I/R-induced brain injury, where an arsenal of ROS are generated by malfunctioning mitochondria, infiltrated neutrophils, and activated microglia (Lalkovičová and Danielisová, 2016).

Mounting evidence indicates that overproduction of ROS *via* microglial NADPH oxidase (NOX), as well as reactive nitrogen species (RNS), play a critical role in DND following I/R injury (Wang et al., 2006). NOX comprises six subunits; membrane-bound (gp91^phox^ and p22^phox^), cytosolic subunits (p40^phox^, p47^phox^, and p67^phox^), and low-molecular weight GTPase Rac (Rastogi et al., 2016). Previous studies reported that administration of apocynin, a well-known NOX inhibitor, attenuated microglial activation and neuronal death (Qin et al., 2017).

Apart from OS, activation of microglia and astrocyte is also associated with enhancement of inflammatory reactions with increased neuronal expression of the redox-sensitive nuclear factor kappa B (NF-κB). This transcription factor empowers the generation of proinflammatory enzymes and cytokines, including tumor necrosis factor-α (TNF-α), inducible nitric oxide synthase (iNOS), and intracellular adhesion molecule-1 (ICAM-1) (Zhang et al., 2016). These events amplify the inflammatory cascade and trigger the recruitment of neutrophils, thereby exacerbating the ischemic insult (Cheng and Lee, 2016; Sapkota et al., 2017).

On the molecular level, constellation of signaling pathways intersects to augment neuronal damage following ischemic insult. One of the signaling cue that has a crucial impact on endorsing cell survival after I/R is the phosphatidylinositol 3-kinase (PI3K)/protein kinase B (Akt) signaling pathway (Qu et al., 2015). Activated PI3K phosphorylates its downstream target Akt to abate cell death through the phosphorylation/inactivation of its downstream substrates, *viz*, pro-apoptotic proteins B-cell lymphoma 2 (Bcl-2)- associated death protein (BAD), c-Jun N-terminal kinase (JNK), and class O members of the forkhead transcription factor family (FOXOs) (Liu et al., 2010; Zhao et al., 2016).

The FOXO family consists of various members including FOXO1A, FOXO3A, and FOXO4 (Fukunaga and Shioda, 2010). Among them, FOXO3A has been recognized to regulate neuronal apoptosis by inducing the killer protein Bcl-2 interacting mediator of cell death (Bim) and Fas ligand (Shioda et al., 2007). Previous studies showed that suppression of Akt activity results in dephosphorylation of FOXO3A leading to its nuclear translocation and the enhancement of Bim expression. The latter triggers cytochrome *c* release from the mitochondria, caspase-3 activation, and eventually persuading apoptosis (Li et al., 2015).

Aside from their destructive role, ROS/RNS initiate apoptotic signaling pathways, such as JNK3 (Hu et al., 2012), which is an important subclass of the mitogen-activated protein kinase family (Meloni et al., 2014). Activated JNK3 aggravates ischemia-induced apoptotic signaling by promoting the expression/activity of crucial proteins involved in apoptosis, such as c-Jun, Bcl-2 associated X protein (Bax), and caspase-3 (Hetz et al., 2005; Shao et al., 2016). Hence, inhibition of JNK3 shows a neuroprotective effects against several models of cerebral I/R injury (Ge et al., 2017; Luo et al., 2017).

Today the pharmacological actions of statins, competitive inhibitors of 3-hydroxy-3-methylglutaryl coenzyme A (HMG-CoA) reductase, including rosuvastatin (RUS), have been extended far beyond their reputed therapeutic use as anti-hyperlipidemic, where they afford substantial effects in incidences linked to I/R comprising the brain (Prinz et al., 2008). Previously, the neuroprotective effect of RUS has been attested against spinal cord I/R injury (Yavuz et al., 2013), subarachnoid hemorrhage (Uekawa et al., 2014), and traumatic brain injury (Kahveci et al., 2014). The protective effect of RUS relies on the modulation of several signal transduction pathways, such as NF-κB, PI3K/Akt, and JNK (De las Heras et al., 2013; Chang et al., 2015; Liu et al., 2017).

On the other hand, Coenzyme Q10 (CoQ10), is a naturally occurring fat-soluble vitamin like ubiquinone, contained in most cellular membranes including the mitochondria (James et al., 2004). CoQ10 regulates mitochondrial oxidative phosphorylation and consequently ATP production. Additionally, CoQ10 acts as a potent antioxidant either by scavenging ROS, hence, preventing the initiation and dissemination of membrane oxidation, and/or *via* restoring cellular antioxidants, such as α-tocopherol and ascorbic acid (Chen et al., 2017). Moreover, CoQ10 exhibits estimated beneficial effects against experimental cerebral I/R injury (Abd-El-Fattah et al., 2010), diabetes mellitus (Amin et al., 2014), as well as myocardial injury (Mustafa et al., 2017).

Notwithstanding their applications as anti-lipidemic agents, statins have been reported to deplete/or lower the circulating levels of CoQ10 (Mohammadi-Bardbori et al., 2015), since, the cholesterol biosynthesis pathway, which is repressed by these agents is shared by other molecules including CoQ10 (Martin et al., 2011). Deficiency of CoQ10 results in suppression of mitochondrial activity with increased ROS generation and inflammation (Spindler et al., 2009).

Additionally, decreased CoQ10 levels are believed to be a pathological feature of the increased OS in neurodegenerative diseases (Yang et al., 2016), and diabetes (Sourris et al., 2012). Further, studies by Horecky et al. (2011) and Belousova et al. (2016), reported that cerebral I/R reduces brain mitochondrial and plasma levels of CoQ9 and CoQ10, events that are attributed to their consumption *via* ROS generation. Interestingly, these studies also reported that oral supplementation of CoQ10 remarkably blunted the I/R-induced brain injury (Horecky et al., 2011; Belousova et al., 2016). To this end, CoQ10 seems to be an attention-grabbing component that merits supplementation in patients at high risk of stroke and treated with statins.

As far as our team knows, no studies have been performed on the effects of combined treatment with RUS and CoQ10 against cerebral I/R injury. Therefore, the present study was performed to investigate the possible beneficial use of CoQ10 as an adds-on therapy to RUS in a rat model of transient global ischemia. In addition, the study divulged some of the possible signaling pathways involved in the neuroprotective mechanisms of RUS and CoQ10.

## Materials and Methods

### Animals

Adult male Wistar rats, aged 10 weeks and weighing 260–280 g, were used in the current study. The animals were housed under adjusted laboratory conditions (temperature of 24 ± 1°C), humidity (55 ± 5%), and a 12/12 h dark/light cycle. Animals were left for 1 week to accommodate before any experimental procedures and they had free access to standard rat diet chow and tap water.

The present study followed the recommendations of the Guide for the Care and Use of Laboratory Animals published by the US National Institute of Health (NIH, 1996). The protocol was approved by the Research Ethical Committee of the Faculty of Pharmacy, Cairo University (Cairo, Egypt; Permit Number: PT 2110). Surgical procedures and euthanasia were carried out under thiopental anesthesia and sincere efforts were exerted to reduce the suffering of animals.

### Drugs and Chemicals

CoQ10 was obtained from Sigma-Aldrich (St. Louis, MO, United States), whereas rosuvastatin (RUS) and CoQ10 solubilizing agents that include [Labrasol, Labrafil (M1944 CS), and Capryol 90] were generously gifted from the Global Napi for Pharmaceutical industry (Cairo, Egypt) and Gattefosse (Lyon, France), respectively.

### Preparation of Solubilized CoQ10

CoQ10 has poor water solubility and bioavailability, hence, after careful consideration and consulting, CoQ10 self-emulsifying drug delivery system (SEDD) formulation was prepared by dissolving an amount of CoQ10 (6% w/v) in a mixture of Labrasol (surfactant, 61.1%), Labrafil M1944 CS (oil, 23.5%), and Capryol 90 (co-surfactant, 9.4%) at 60°C in an isothermal water bath. The final mixture was continuously stirred with magnetic bar until a clear solution was obtained; the usefulness of the SEDDs over the commercially available formulations was confirmed previously (Balakrishnan et al., 2009). The surfactants used in these formulations are known to enhance the bioavailability *via* improving drug dissolution and increasing intestinal epithelial permeability. In addition, the long-chain oil, Labrafil M1944 CS has been reported to improve lymphatic absorption (Caliph et al., 2000).

Since no significant difference was detected between sham and I/R groups receiving saline, the RUS vehicle, and those receiving the CoQ10 solubilizing agents, after performing a preliminary study, therefore, the results of the current study were compared to the sham and I/R control groups receiving the saline.

### Rat Model of Transient Global Cerebral I/R

Transient global cerebral ischemia was induced according to the method annotated previously (Collino et al., 2006). In short, rats were anesthetized with thiopental sodium (30 mg/kg, i.p.), a ventral midline incision was made to expose both common carotid arteries (CCAs). After careful isolation from vagus nerve and surrounding tissues, ischemia was initiated by bilateral ligation of the CCAs by the mean of non-traumatic aneurysmal clips. After 60 min of the occlusion, the clips were gently removed to assist reperfusion for 24 h or 5 days. Rectal temperature was adjusted at 37°C *via* an overhead heating lamp. The incision was then stitched using silk suture and sprayed by a local anesthetic and antibiotic. A dose of meloxicam (1 mg/kg, S.C) (Basrai et al., 2016) was used to relief animal pain. Sham-operated animals were treated identically, except that CCAs were not occluded.

### Experimental Design and Treatment Protocol

In this study, a final of 90 rats were assigned into three sets, each set comprised five groups (*n* = 6); in all sets the first group served as the sham-operated control and the second one was the I/R control group (in this group dead animals were 2-3/9). In groups 3, 4, and 5 rats were pretreated with RUS (10 mg/kg; Ma et al., 2013), CoQ10 (10 mg/kg; Kalayci et al., 2011), and RUS + CoQ10, respectively. All treatment regimens were administered p.o., for 7 days and I/R was induced on day 8.

### Tissue Collection and Preparation

One day after reperfusion, animals in the first two sets were sacrificed by an overdose of thiopental, and the brains were immediately harvested and the two hippocampi were dissected on ice cold plates. In the 1st set, the two hippocampi/rat were used for the determination of redox biomarkers and ELISA measurements. In the 2nd set, one hippocampus/rat/group (30 hippocampi) was used for parameters analyzed by Western blot technique, whereas the second half was submerged overnight in RNA later solution for the subsequent quantification of hippocampal gene expression using quantitative real-time polymerase chain reaction (qPCR).

On the other hand, animals in the third set were sacrificed 5 days post I/R, and used for histological assessment of hippocampal DND. In this set, animals were subjected to transcardiac perfusion using paraformaldehyde in phosphate buffer saline (4%) solution then the brains were rapidly removed and immersed in 10% formaldehyde to be embedded in paraffin. After processing, the coronal sections (4–5 μm thick) at the level of dorsal hippocampus were selected and processed for hematoxylin and eosin (H&E) staining.

### Histological Analysis

The degree of hippocampal injury was assessed by counting the number of viable neurons in the hippocampal CA1 area (x400) using computerized image-analyzer (Leica Qwin 500, Cambridge, United Kingdom). Neurons exhibiting visible nuclei, clear nucleoplasm, and distinctive nucleolus were counted. The mean number of CA1 neurons per mm was calculated in two successive sections/hippocampus/rat (6 rats/group). To avoid bias, histological assessment was performed by an investigator who was unaware of the condition of each specimen.

### Biochemical Analysis

#### Estimation of Oxidative Biomarkers and Cellular Defense

Hippocampal tissue was homogenized in 10 volumes of chilled phosphate buffer (pH 7.4) in a glass manual homogenizer. The homogenate was centrifuged at 10,000 × *g* for 20 min at 4°C. The resulting supernatant was used for the estimation of the following parameters using the corresponding assay kit as shown in parenthesis: malondialdehyde (MDA) (Bio-diagnostic, Egypt), reduced glutathione (GSH) (Cell Biolabs, San Diego, CA, United States, Cat. # STA-312), and superoxide dismutase (SOD) activity (Trevigen, Gaithersburg, Germany, Cat. # 7500-100-K). The above stated biomarkers were processed according to manufacturers’ procedures.

#### Estimation of Inflammatory Cytokines and Apoptotic Markers

The parameter, its corresponding kit, and its source are displayed as follows: TNF-α (Ray Biotech, Norcross, GA, United States, Cat. # ELR-TNFalpha-001C), rat ICAM-1 (EIAab, Wuhan, China, Cat. # E0048r), total nitric oxide (NOx) (Assay Designs, Ann Arbor, MI, United States, Cat. No. 917-010), and caspase-3 activity (ApoTarget, Invitrogen, Carlsbad, CA, United States, Cat. # KHZ002). The aforementioned biomarkers were assessed according to the designated manufacturers’ instructions.

#### Western Blot Analysis

Briefly, the nuclear, cytoplasmic, and mitochondrial proteins of the hippocampus were extracted by NE-PER Nuclear and Cytoplasmic Extraction Kit (Thermo Scientific Co., Hanover Park, IL, United States) and Mitochondrial Fractionation Kit (Abcam, United States) conferring to manufacturer’s guidelines. Protein concentration in the hippocampus lysate was estimated using BCA protein assay kit (Bio-Rad, Hercules, CA, United States). Protein samples (30–50 μg per lane) were separated by SDS-PAGE then transferred into nitrocellulose membrane. The membranes were blocked with 5% (w/v) non-fat dry milk in Tris buffered saline-Tween 20 (0.025 M Tris; 0.15 M NaCl; 0.05% Tween 20; pH 7.4) and incubated with primary antibodies overnight at 4°C. The following primary antibodies [mouse monoclonal antibodies against Akt (1:1000), *p*-Akt (Ser473,1:1000), Bcl-2 (1:200), JNK3 (1:1000), *p*-JNK3 (1:1000), and *p*-c-Jun (1:200) were purchased from Santa Cruz Biotechnology (Dallas, TX, United States), whereas antibodies against Bax (1:100), Bim (Rabbit ployclonal, 1:1000), c-Jun (Mouse monoclonal, 1:2000), cytochrome *c* (Mouse monoclonal, 1:200), FOXO3A (Rabbit polyclonal, 1:1000), *p*-FOXO3A (Rabbit polyclonal, 1000), MPO (Rabbit polyclonal, 1:200), NF-κB p6 (Rabbit polyclonal, 1:1000), iNOS (Rabbit polyclonal, 1:200), and β-actin (Mouse monoclonal 1:1000) were procured from Thermo Scientific Co. (Hanover Park, IL, United States). The next day, the membranes were washed and incubated with secondary antibodies (Thermo Scientific Co., Hanover Park, IL, United States) for 1 h at 25°C. The optical densities of the expressed proteins were analyzed by ChemiDoc^TM^ imaging system using Image Lab^TM^ software version 5.1 (Bio-Rad Laboratories Inc., Hercules, CA, United States). The results were expressed as arbitrary units after normalization for β-actin protein expression.

#### Real-time Quantitative Polymerase Chain Reaction (RT)-PCR

Hippocampal p47^phox^ and gp91^phox^ gene expression were determined using qt-PCR analysis. Total RNA was extracted using RNA easy (Mini Kit, QIAGEN, United States) in accordance with the manufacturer’s instructions. cDNA was synthesized from extracted RNA using Reverse Transcriptase Kit (RT Kit, Thermo Scientific, United States). To evaluate the expression of target genes, RT-PCR was performed using StepOnePlus^TM^ (Applied Biosystem, Foster City, CA, United States) with SYBR Green PCR Master Mix (Applied Biosystems, Foster City, CA, United States) in a 25-μl reaction with 900 nM primers possessing the following sequences: β-actin sense :5′-CCTTCCTGGGCATGGAGTCCT-3′; antisense :5′-GGAGCAATGATCTTGATCTTC-3′, p47^phox^ sense: 5′-GTC GTGGAGAAGAGCGAGAG-3′; antisense: 5′-CGC TTTGATGGTTACATACGG-3′, and gp91^phox^ sense primer 5′-CCG TATTGTGGGAGACTGGA-3′; antisense: 5′-CTTGAGAATGGAGGCAAAGG-3′. Amplification conditions were: 2 min at 50°, 10 min at 95° and 40 cycles of denaturation for 15 s and annealing/extension at 60° for 10 min. Data from real-time analysis were calculated using the v1⋅7 sequence detection software from PE Biosystems (Foster City, CA, United States). Relative expression of examined gene mRNA was estimated using the comparative Ct method. All values were normalized to β-actin which was used as the control housekeeping gene.

### Statistical Analysis

Values were expressed as mean of six rats ± SEM, and statistical analyses were performed using one-way analysis of variances (ANOVA) followed by Tukey’s *post hoc* Multiple Comparisons among treatment means. The analysis was performed using GraphPad Prism software (version 5.0; GraphPad Software, Inc., San Diego, CA, United States). Differences were considered significant at *p* < 0.05.

## Results

### Effect of RUS and/or CoQ10 on I/R-Induced Hippocampal Structure Damage

**FIGURE 1:**
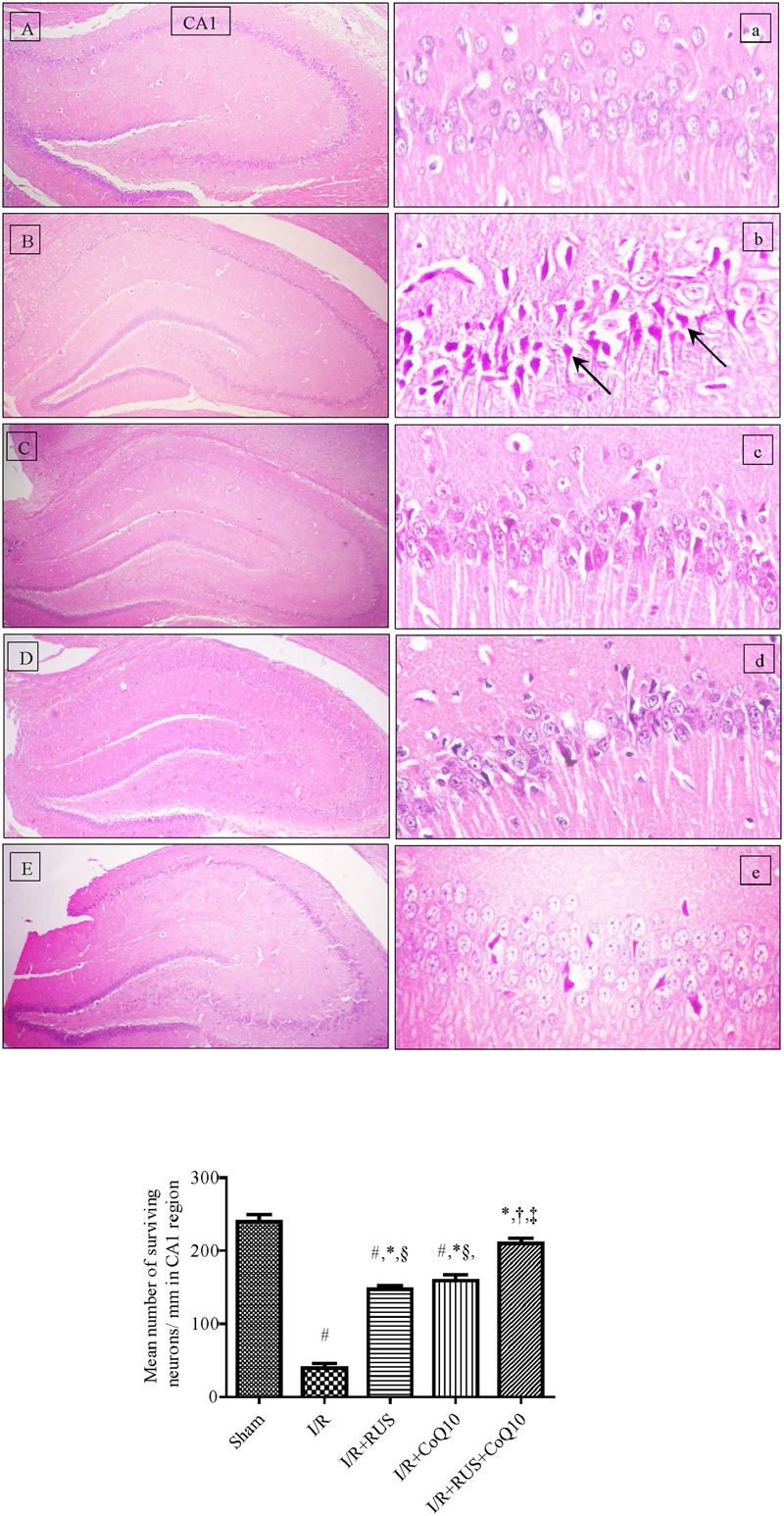
Descriptive images of H&E staining (top) displaying the neuroprotective effect of Rosuvastatin and/or CoQ10 on hippocampal CA1 area. **(A,a)** Sham group, **(B,b)** I/R, **(C,c)** I/R+RUS, **(D,d)** I/R+CoQ10, and **(E,e)** I/R+RUS+CoQ10. Histological examinations were achieved 5 days following 60 min ischemia. Arrows point to damaged neurons (x400). Lower panel indicates the mean number of salvaged neurons per mm in the CA1 subfield. Rosuvastatin (RUS; 10 mg/kg), CoQ10 (10 mg/kg), and their combination were administered p.o. for 7 days before ischemic insult. Values are presented as mean (*n* = 6) ± SEM. Statistical analysis was carried out using one-way ANOVA followed by Tukey’s *post hoc* multiple comparison test. As compared with (#) sham-operated, (^∗^) I/R, (†) RUS, (‡) CoQ10, and (§) combination pretreated groups (*P* < 0.05).

Photomicrographs of (B, b) I/R sections showed morphological aberrations described as a selective and widespread neuronal degeneration in the CA1 area of the hippocampus, 5 days after 60 min ischemia compared to the (A, a) normal sham operated group. Various neurons with eosinophilic shrunken cytoplasm and pyknotic nuclei [black arrow] were observed. Pre-ischemic administration of (C, c) RUS, notably hindered these alterations, in comparison to the I/R group. Similarly, (D, d) pre-administration of CoQ10 hampered the injurious insult on CA1 neurons, and a better effect was detected in the (E, e) RUS + CoQ10 treated group. Validating the results of the histological findings (**Figure 1**, lower), showed a reduction in the number of viable neurons in the CA1 subfield of the ischemic group, compared to the sham one; however, RUS-treated group depicted a substantial restoration in the number of salvaged neurons at the same area of interest. These impacts were closely similar to those afforded by CoQ10. Meanwhile, combining RUS with CoQ10 achieved maximal benefits as evidenced by the remarkable enhancement of the mean number of rescued neurons per mm in the same affected area compared to either RUS or CoQ10.

### Effect of RUS and/or CoQ10 on Oxidative Stress Parameters after I/R Insult

**FIGURE 2:**
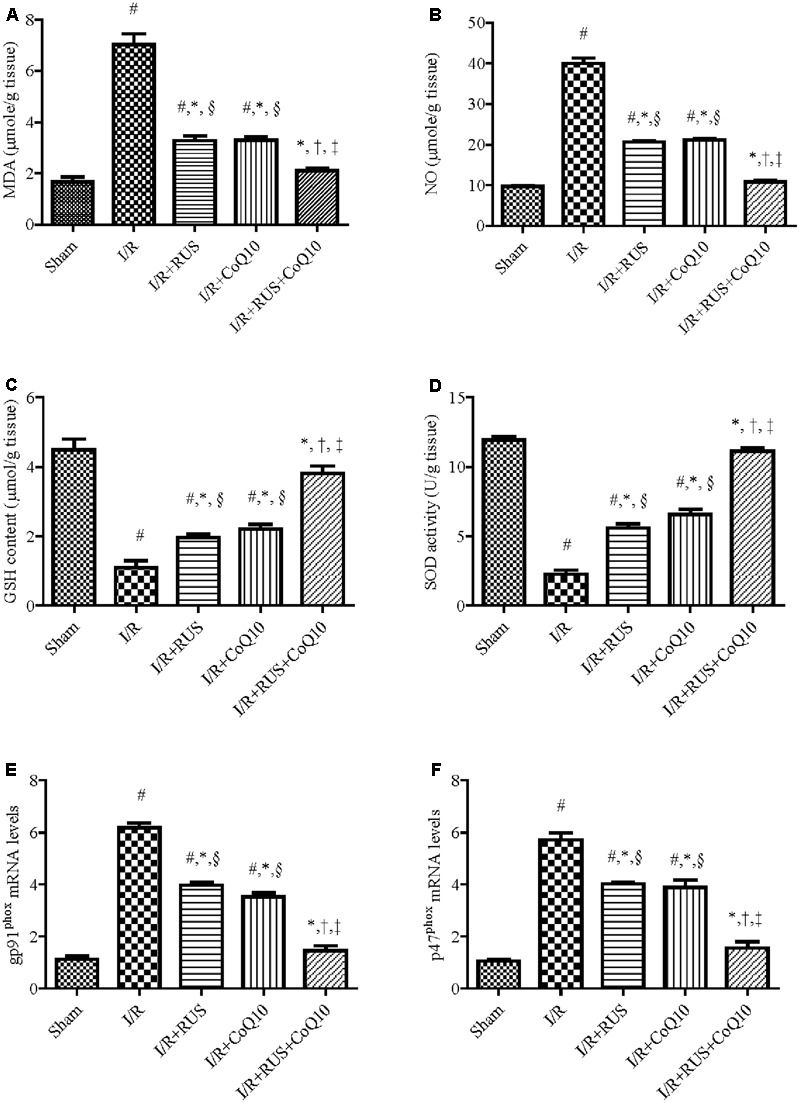
Modulatory effects of Rosuvastatin and/or CoQ10 on **(A)** MDA, **(B)** NO, **(C)** GSH, **(D)** SOD, and **(E)** mRNA expression of gp91^phox^ and **(F)** p47^phox^ subunits. Measurements were achieved 24 h following 60 min ischemia. Rosuvastatin (RUS; 10 mg/kg), CoQ10 (10 mg/kg), and their combination were administered p.o. for 7 days before ischemic insult. Values are presented as mean (*n* = 6) ± SEM. Statistical analysis was carried out using one-way ANOVA followed by Tukey’s *post hoc* multiple comparison test. As compared with (#) sham-operated, (^∗^) I/R, (†) RUS, (‡) CoQ10, and (§) combination pretreated groups (*P* < 0.05).

I/R markedly increased OS (**Figure 2**), as indicated by the high contents of MDA and NO in parallel with a decline in the defense molecules GSH and SOD, when compared to the sham group. Pre-ischemic administration of RUS significantly protected against OS, by lowering MDA, NO and replenishing GSH and SOD. In the same context, RT-PCR examination revealed a marked upregulation of mRNA expression of NOX subunits, gp91^phox^ and p47^phox^ in the I/R group, effects that were markedly mitigated by RUS and CoQ10 pre-administration. It is worth mentioning that the combination effect maintained these parameters at their normal values.

### Effect of RUS and/or CoQ10 on Inflammatory Mediators after I/R Insult

**FIGURE 3:**
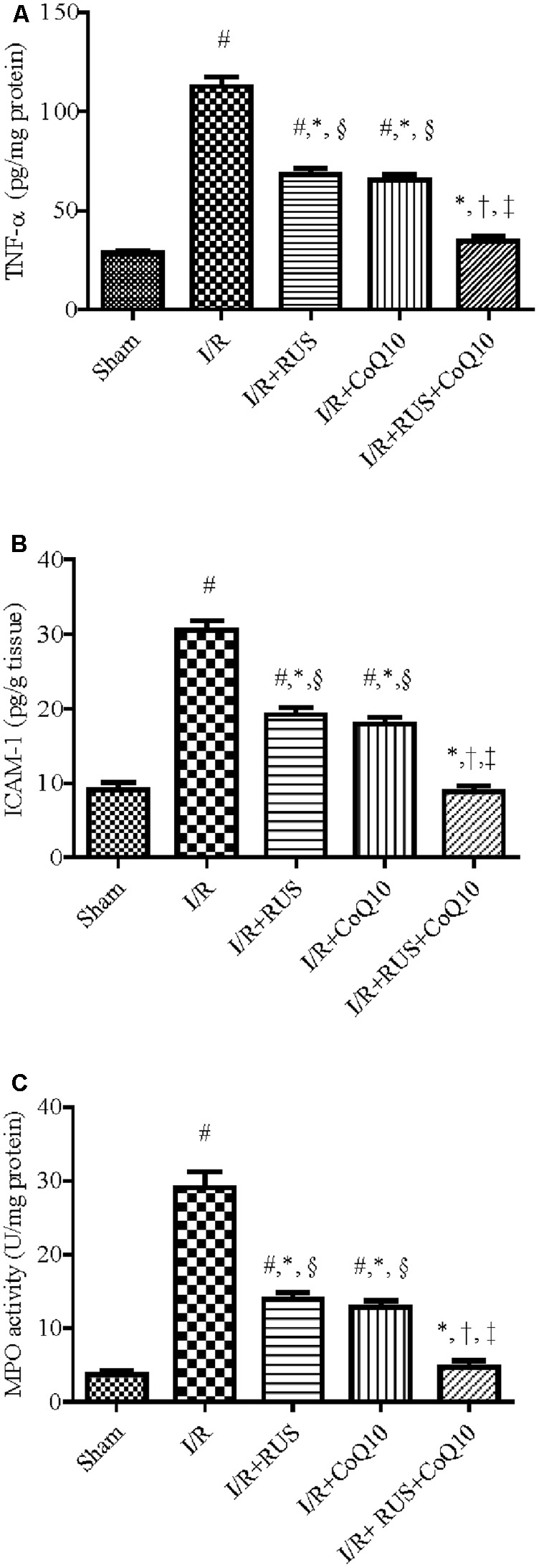
Rosuvastatin and/or CoQ10 attenuate inflammatory markers, **(A)** TNF-α, **(B)** ICAM-1, and **(C)** MPO activity. Measurements were achieved 24 h following 60 min ischemia. Rosuvastatin (RUS; 10 mg/kg), CoQ10 (10 mg/kg), and their combination were administered p.o. for 7 days before ischemic insult. Values are presented as mean (*n* = 6) ± SEM. Statistical analysis was carried out using one-way ANOVA followed by Tukey’s multiple comparison test. As compared with (#) sham-operated, (^∗^) I/R, (†) RUS, and (‡) CoQ10, and (§) combination pretreated groups (*P* < 0.05).

**FIGURE 4:**
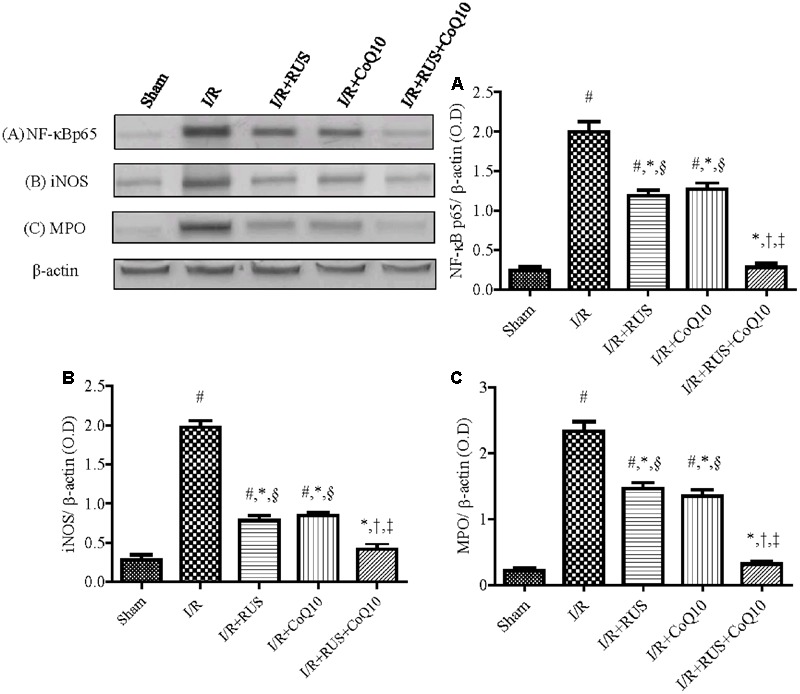
Rosuvastatin and/or CoQ10 downregulate the hippocampal protein expression of activated NF-κBp65, iNOS, and MPO in rats subjected to transient ischemia. Representative Western blots and optical densities of **(A)** NF-κBp65, **(B)** iNOS, and **(C)** MPO. Measurements were achieved 24 h following 60 min ischemia. Rosuvastatin (RUS; 10 mg/kg), CoQ10 (10 mg/kg), and their combination were administered p.o. for 7 days before ischemic insult. Values are presented as mean (*n* = 6) ± SEM. Statistical analysis was carried out using one-way ANOVA followed by Tukey’s *post hoc* multiple comparison test. As compared with (#) sham-operated, (^∗^) I/R, (†) RUS, and (‡) CoQ10, and (§) combination pretreated groups (*P* < 0.05).

As depicted in **Figure 3**, I/R caused a surge of inflammatory mediators, manifested by a notable increase in the hippocampal content/activity of TNF-α, ICAM-1, and MPO compared to the sham group. Additionally, Western blot analysis revealed an extensive hippocampal expression of NF-κB p65, iNOS, and MPO in rats exposed to I/R (**Figure 4**). These events were significantly abrogated by the pre-ischemic administration of RUS and CoQ10, with their combination mediating responses that surpassed either treatment alone.

### Effect of RUS and/or CoQ10 on Akt, FOXO3A, and Bim Signaling Transduction after I/R Insult

**FIGURE 5:**
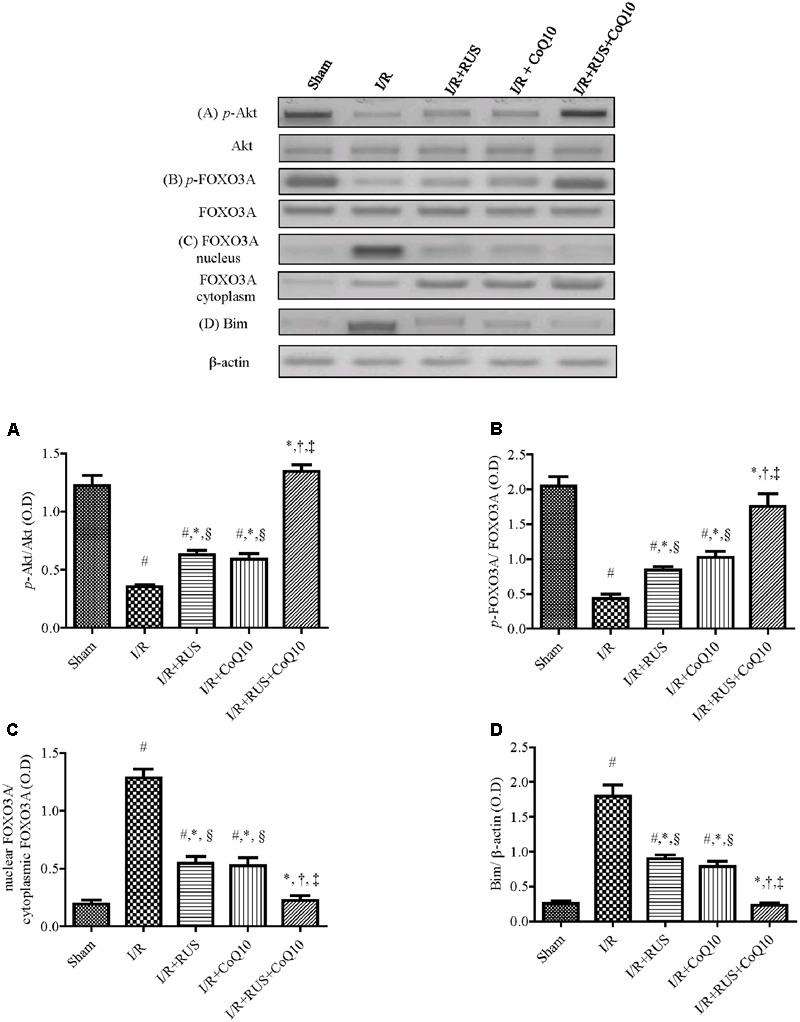
Rosuvastatin and/or CoQ10 upregulate the hippocampal protein expression of *p*-Akt and *p*-FOXO3A with the decline of FOXO3A nuclear translocation and Bim. Representative Western blots and optical densities of **(A)** *p*-Akt, **(B)** *p*-FOXO3A, **(C)** nuclear FOXO3A, and **(D)** Bim in rats subjected to transient ischemia. Measurements were achieved 24 h following 60 min ischemia. Rosuvastatin (RUS; 10 mg/kg), CoQ10 (10 mg/kg), and their combination were administered p.o. for 7 days before ischemic insult. Values are presented as mean (*n* = 6) ± SEM. Statistical analysis was carried out using one-way ANOVA followed by Tukey’s *post hoc* multiple comparison test. As compared with (#) sham-operated, (^∗^) I/R, (†) RUS, and (‡) CoQ10, and (§) combination pretreated groups (*P* < 0.05).

Compared to sham animals, **Figure 5** revealed that global ischemia abated the protein expression of *p*-Akt and *p*-FOXO3A, while it increased that of the translocated nuclear FOXO3A to augment Bim. Pretreatment regimens averted the I/R-induced dephosphorylation of Akt and FOXO3A and lessened the protein expression of Bim and nuclear FOXO3A. These effects were more pronounced in the combination group when compared to individual treatment groups.

### Effect of RUS and/or CoQ10 on JNK3 and c-Jun Signaling Pathway after I/R Insult

**FIGURE 6:**
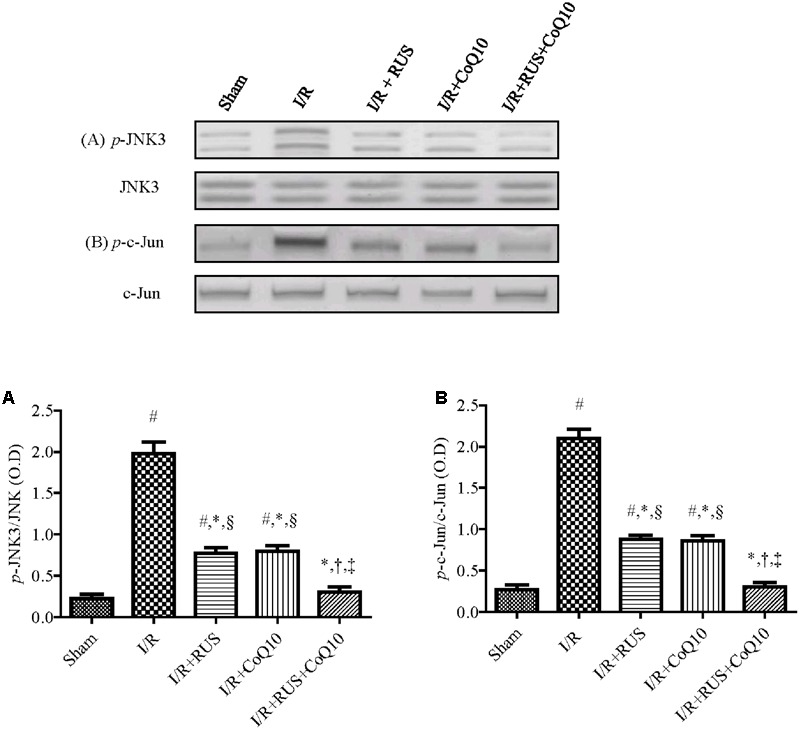
Rosuvastatin and/or CoQ10 mitigate the hippocampal protein expression of *p*-JNK3 and *p*-c-Jun. Representative Western blots and optical densities of **(A)** *p*-JNK3 and **(B)** *p*-c-Jun in rats subjected to transient ischemia. Measurements were achieved 24 h following 60 min ischemia. Rosuvastatin (RUS; 10 mg/kg), CoQ10 (10 mg/kg), and their combination were administered p.o. for 7 days before ischemic insult. Values are presented as mean (*n* = 6) ± SEM. Statistical analysis was carried out using one-way ANOVA followed by Tukey’s *post hoc* multiple comparison test. As compared with (#) sham-operated, (^∗^) I/R, (†) RUS, and (‡) CoQ10, and (§) combination pretreated groups (*P* < 0.05).

Western blot analysis divulged that I/R promoted JNK3 signaling cascade (**Figure 6**) evidenced by the enhanced phosphorylation of JNK3 and c-Jun without affecting the corresponding total protein content, compared to the control group. These changes were halted by the three treatments intervention. Again, the combination group showed the best effects compared to each drug alone.

### Effect of RUS and/or CoQ10 on Apoptotic Biomarkers after I/R Insult

**FIGURE 7:**
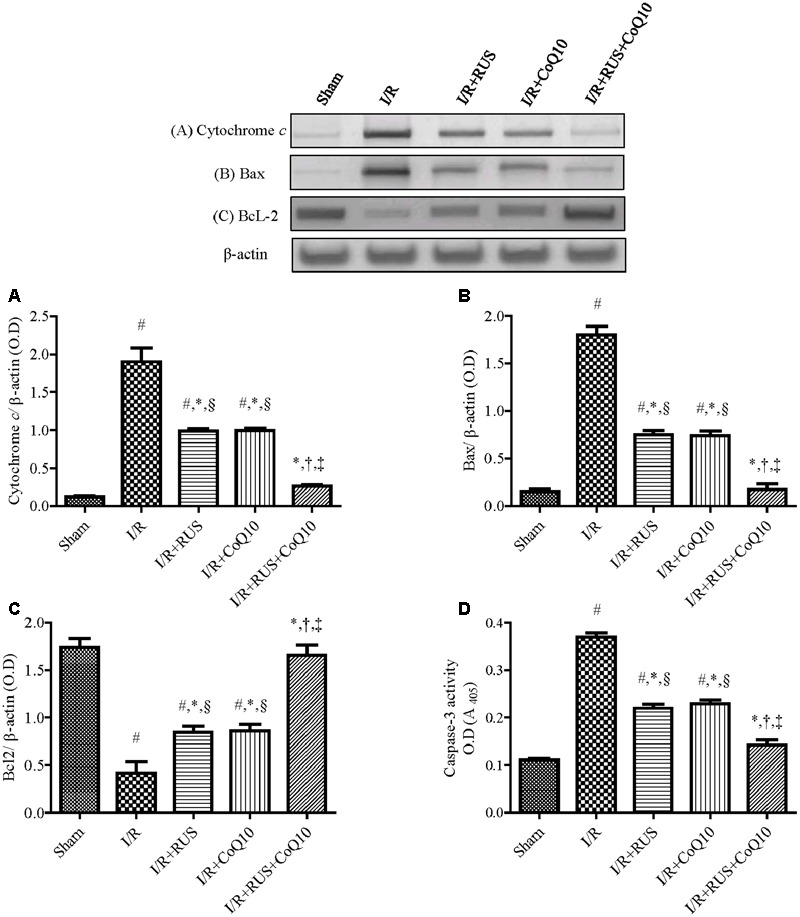
Rosuvastatin and/or CoQ10 abate hippocampal cytochrome *c*, Bax, and caspase-3 activity with the enhancement of Bcl2 levels. Representative Western blots and optical densities of **(A)** Cytochrome *c*, **(B)** Bax, **(C)** Bcl2, and **(D)** caspase-3 activity in rats subjected to transient ischemia. Measurements were achieved 24 h following 60 min ischemia. Rosuvastatin (RUS; 10 mg/kg), CoQ10 (10 mg/kg), and their combination were administered p.o. for 7 days before ischemic insult. Values are presented as mean (*n* = 6) ± SEM. Statistical analysis was carried out using one-way ANOVA followed by Tukey’s *post hoc* multiple comparison test. As compared with (#) sham-operated, (^∗^) I/R, (†) RUS, (‡) CoQ10, and (§) combination pretreated groups (*P* < 0.05).

As shown in **Figure 7**, transient global cerebral ischemia triggered apoptotic-killing machinery in hippocampal tissues as manifested by the elevation of cytochrome *c*, pro-apoptotic Bax, and caspase-3 activity, along with a reduction in the anti-apoptotic Bcl-2 contents, when compared to the sham group. All treatments used offset these alterations in favor of cell survival, with the superior effect mediated by the combination regimen.

The original western blot images for **Figures 4–7** are supplied as Supplementary Material entitled (Supplementary Figures 1–4).

## Discussion

**FIGURE 8:**
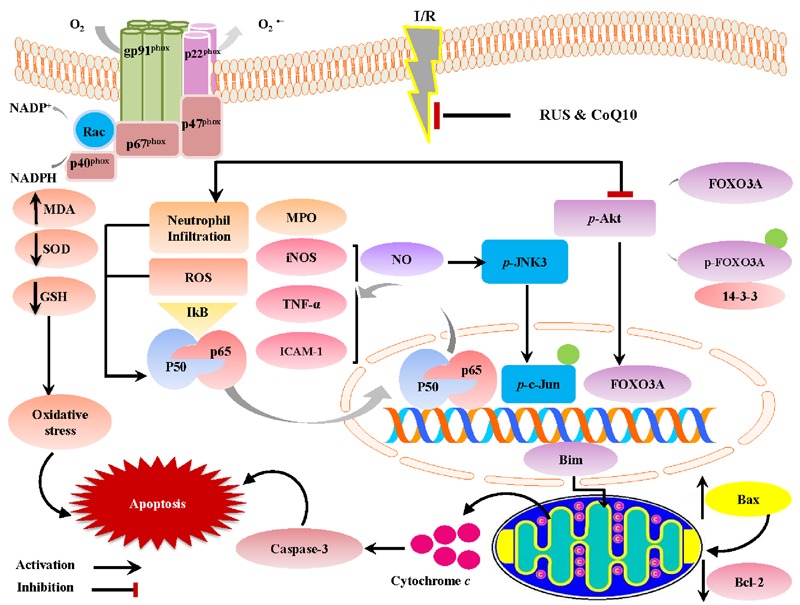
A proposed framework illustrating the versatile impacts of RUS and CoQ10 against I/R-induced hippocampal injury.

The current investigation sheds light on the neuroprotective potentials of RUS in a rat model of I/R-induced brain injury. These favorable outcomes were mediated *via* the suppression of oxidative/nitrosative stress and inflammation, chiefly through the inhibition of NF-κBp65. Our results are extended to reveal its anti-apoptotic action that can be linked to the modulation of Akt/FOXO3A/Bim and JNK/c-Jun/Bax signaling pathways. Interestingly, the beneficial effects of RUS were analogous to those exerted by CoQ10 (**Figure 8**).

Although RUS and CoQ10 confer their neuroprotection against various neurodegenerative models, yet the current study is the first to address the effect of RUS in a global ischemia model and to provide new machineries that may emphasize its neuroprotective effects. The study also supports the usefulness of using CoQ10 as an adds-on supplement with statin drugs.

The contribution of OS in the pathogenesis of ischemia-induced hippocampal injury has been explored by numerous studies (Chen et al., 2014; Ma et al., 2017). Data of the current work revealed the antioxidant and free radical scavenging capacities of RUS; the statin reduced hippocampal MDA and elevated the content/activity of defense molecules, GSH and SOD. These findings are in agreement with the reported effect of RUS against diabetes-induced testicular damage (Heeba and Hamza, 2015) and spinal cord I/R (Die et al., 2010; Ucak et al., 2011). As a possible mechanism, RUS downregulated the expression of p47^phox^ and gp91^phox^, subunits of NOX, which is a source of ROS production; this finding concurs with that in a previous study testing RUS against focal cerebral ischemia (Ma et al., 2013). In the same context (Qin et al., 2017) reported that pharmacological inhibition of NOX or genetic deletion of gp91^phox^ or p47^phox^, improved neuronal survival following reoxygenation to endorse the role of this system in I/R insult. Thus, inhibiting NOX may represent an important mechanism that underlies the RUS neuroprotective effect against oxidative damage during global cerebral I/R injury.

Similarly, the current results support the known anti-oxidant capacity of CoQ10 to reduce OS in hippocampal tissue, as manifested herein by the replenishment of the endogenous antioxidants, *viz.*, GSH and SOD, and leveling MDA off, besides the suppression of NOX subunits, results that are in harmony with that in different models (Sohet et al., 2009; Tsai et al., 2011). Nevertheless, combining both agents showed the best antioxidant/free radical scavenging effects keeping the aforementioned parameters within the normal level.

Moreover, RUS and CoQ10 alone or in combination opposed the I/R-induced upregulation of hippocampal iNOS expression and elevation of NO to support previous findings (Choi et al., 2012; Park et al., 2012). Earlier reports indicated that I/R activates microglial cells containing iNOS and gp91^phox^ (Jin et al., 2010). Once activated, microglia generate surplus levels of NO that undermines hippocampal CA1 neuronal viability *via* its conjugation with superoxide anion and the formation of the potent free radical peroxynitrite (Yenari et al., 2010). Indeed, the ability of RUS and CoQ10 to mitigate NO, as well as iNOS, besides the expression of NOX subunits can be linked to the inhibition of the nuclear translocation of NF-κBp65, hence, linking OS with the inflammatory pathways (Gauss et al., 2007).

In the current work, RUS verified its anti-inflammatory effect, which was documented previously (Uekawa et al., 2014), by inhibiting content/activity of TNF-α, ICAM-1, and MPO, a surrogate marker of neutrophils infiltration to support earlier findings in different models (Mayanagi et al., 2008; Kahveci et al., 2014). These impacts can be attributed to the observed downregulation of the transcription factor NF-κB, which has been reported to upregulate the pro-inflammatory cytokine TNF-α (de Oliveira Ferreira et al., 2016). The latter is known to trigger leucocyte recruitment *via* increasing the expression ICAM-1 following ischemic insult (Hou et al., 2010). Our study also illustrated the ability of CoQ10 to suppress the aforementioned inflammatory events triggered by cerebral I/R (Shi et al., 2011; Simão et al., 2012).

These results matched with former studies indicating the anti-inflammatory character achieved by CoQ10 in experimental models of hepatic (Fouad and Jresat, 2012), renal (Fouad et al., 2011), myocardial (Mustafa et al., 2017), and cartilage injury induced by intra-articular injection of monosodium iodoacetate (Lee et al., 2013). Hence, the anti-inflammatory effects of RUS and CoQ10 can signify partly their neuroprotective capacity, since immense evidence elucidates that neuronal death following I/R is linked with an inflammatory response, including infiltration of neutrophils and the release of inflammatory mediators (Shi et al., 2011; Simão et al., 2012).

These facts tone with our observations in which, I/R provoked a state of inflammation proved by an activation of NF-κBp65, which in turn increased hippocampal TNF-α to enhance the protein expression of ICAM-1. The latter was convoyed with the activation and protein expression of MPO, proved herein. Invasion of neutrophils triggers OS *via* the release of ROS, RNS, as well as hypochlorous acid, a powerful cytotoxic oxidant generated by MPO (Bao et al., 2004).

At the molecular level, the studied signal transductions revealed that I/R significantly leveled off the protein expression of *p*-Akt (active) and *p*-FOXO3A (inactive). The latter was associated with the translocation of the free active FOXO3A to the nucleus, as evidenced by its increased nuclear protein expression, to increase that of the pro-apoptotic protein Bim. These findings coincide with previous works (Fukunaga et al., 2005; Zhan et al., 2010), where increased oxidative/nitrosative stresses diminish *p*-Akt expression (Crack and Taylor, 2005) by activating its inhibitor, to inhibit its phosphorylation (Liu et al., 2010).

On the other hand, RUS asserted its protective effect by opposing the I/R-induced dephosphorylation of Akt and FOXO3A, inhibiting its translocation to the nucleus, and thereby hindering the expression of its down-stream target molecule Bim. This pro-apoptotic molecule is a chief mediator of neuronal apoptosis in neonatal ischemia/hypoxia models (Shioda et al., 2007; Li et al., 2015). Enhancement of Akt phosphorylation has been reported to prevent neuronal apoptosis *via* the downregulation of FOXO3A/Bim axis in response to hypoxic/ischemic brain injury (Li et al., 2009; Miyawaki et al., 2009). RUS effect coincides with the results of the *in vitro* study of Zhang et al. (2013), who indicated that RUS protects the adipose-derived mesenchymal stem cells transplanted into infarcted murine hearts against hypoxia/serum deprivation injury *via* the modulation of PI3K/Akt/FOXO3A/Bim signaling pathway.

Although there are no previous studies interpreting the effect of CoQ10 on *p*-FOXO3A, we can assume that CoQ10 induced *p*-FOXO3A could be attributed to the increased phosphorylation of Akt along with the antioxidant capacity of CoQ10, proven herein and supported by the study of Song et al. (2015). These authors found that administration of GSH attenuate cerebral infarct volume in rats exposed to focal ischemia, improved the survival of brain endothelial cells, and reduced FOXO3A nuclear translocation by promoting PI3K/Akt pathway.

Besides, rebuilding the I/R-induced perturbation of Akt as a survival pathway, RUS and CoQ10 extended their activity to entail the pro-death signal pathway JNK, as well. The current data revealed that I/R significantly induced phosphorylation of JNK3 and c-Jun in hippocampal tissue, which are in coherence with previous investigations (Heurteaux et al., 1993; Pei et al., 2016). These events were markedly abrogated by the three regime interventions, which underlie the modulation of JNK signaling pathway as a putative tool for neuroprotection against I/R injury. These results are consistent with the reported inhibitory effect of RUS on *p*-JNK expression in a rat model of cyclosporine-induced nephropathy (Nam et al., 2013) and the ability of CoQ10 to attenuate angiotensin II-induced upregulation of *p*-JNK in human umbilical vein endothelial cells (Tsuneki et al., 2013).

The attenuation of hippocampal *p*-JNK3 could be ascribed to the observed suppression of oxidative/nitrosative stress, since NO derived from iNOS has been reported to enhance JNK3 phosphorylation *via* S-nitrosylation triggering, thus, neuronal cell death. Moreover, administration of AMT, a selective inhibitor of iNOS, ameliorated I/R-induced hippocampal neuronal death through the suppression of *p*-JNK (Pei et al., 2008). Hence, RUS and CoQ10 by inhibiting iNOS, as represented in this work, can clarify the decreased *p*-JNK3. Previous studies defend our data, in which RUS (Die et al., 2010) and CoQ10 (Aboul-Fotouh, 2013; Ulla et al., 2017) inhibited the level of NO and expression of iNOS in various animal models, indicating that both treatments could protect neuronal death through their antioxidant properties with the subsequent inhibition of JNK signaling cue.

Furthermore, another explanation can be led by the upregulation of *p*-Akt, where Shao et al. (2016) reported that administration of LY294002, a PI3K inhibitor, reversed the hippocampal protective effect of atorvastatin, another HMG-CoA reductase suppressor, through the inhibition of *p*-Akt and the upregulation of *p*-JNK3, suggesting the inverse correlation between the two molecules.

In agreement with a recent study (Pei et al., 2016), I/R insult provoked selective neuronal degeneration in the hippocampal CA1 area. This was confirmed by the decrease in the number of salvaged neurons, as compared to the non-ischemic group and can be explained partly by the activation of apoptotic machinery evidenced herein by upregulation of cytochrome *c*, Bax, and caspase-3 with the decline of Bcl-2 levels. These data matched previous studies (Endo et al., 2006a; Abd El-Aal et al., 2013; Yin et al., 2013). The present study affirmed the anti-apoptotic characters of RUS and CoQ10 designated herein by the dampening of hippocampal death, pro-apoptotic markers, and the enhancement of Bcl-2 expression. Indeed, the effects exerted by the combination regimen superseded that mediated by either agent when used alone.

These results are in line with previous reports describing the anti-apoptotic capacity of RUS against apoptosis in contrast media-induced renal injury (Deng et al., 2015), cardiac arrest-induced hippocampal injury (Qiu et al., 2017), and spinal cord-induced neuronal death (Die et al., 2010). These observation are also in harmony with studies that clarified the anti-apoptotic potential of CoQ10 in different *in vivo* (Lee et al., 2014), and *in vitro* (Jing et al., 2015; Chen et al., 2017) studies.

The attenuation of hippocampal apoptosis can be accredited to the observed suppression of lipid peroxidation and inflammation, since excessive exposure of hippocampal tissues to ROS and TNF-α has been reported to enhance neuronal apoptosis. Additionally, the rescued Akt cascade to revive the hippocampal CA1neurons can be another explanation for the notable neuroprotection, since increased *p*-Akt phosphorylates and inactivates the transcription factor FOXO3A, hence attenuating its nuclear translocation to alleviate apoptosis driven by Bim-mediating signaling pathway (Shioda et al., 2007). Moreover, *p*-Akt suppresses the mitochondrial release of cytochrome *c* (Hirai et al., 2004) and increases the anti-apoptotic Bcl-2 (Qi et al., 2015; Zhu et al., 2016). The latter also participates in reducing cytochrome *c* (Zhao et al., 2003) and alleviates oxidative damage caused by ROS overexpression (Niture and Jaiswal, 2012) as another mechanism for saving neurons from degeneration. Additionally, activation of Akt efficiently tapers ROS generation by overexpressing SOD (Endo et al., 2006b). All these events are verified in the present work.

However, these findings are just the tip of the iceberg; the ability of RUS/CoQ10 to suppress ROS/RNS may be responsible for neuronal salvage, since these radicals are known to enhance neuronal apoptosis (Gong et al., 2015). These free radicals also activate JNK3 signaling pathway to trigger the expression/activation of pro-apoptotic genes, such as c-Jun, Bim, Bax, which trigger the release of cytochrome *c* and the activation of caspase-3 leading to cell apoptosis (Gao et al., 2005; Pei et al., 2016).

## Conclusion

The present study has verified the neuroprotective potentials of RUS against cerebral global I/R injury by virtue of its versatile actions including suppression of hippocampal OS, inflammation, and apoptosis with the involvement of NF-κBp65/TNF-α/gp91^phox^ and p47^phox^, Akt/FOXO3a/Bim, and JNK3/c-Jun/Bax signaling cues. These impacts were analogous to those of CoQ10, whereas, its combination with rosuvastatin exerted effects that surpassed either treatment alone. The study also appoints CoQ10 as an adds-on therapy with statins.

## Author Contributions

Conceived and designed the experiments: HSE. Performed the experiments: SAE. Analyzed the data: SAE. Contributed reagents/materials/analysis tools: SAE. Wrote the article: HSE and SAE. Critical revision: HSE and MAE.

## Conflict of Interest Statement

The authors declare that the research was conducted in the absence of any commercial or financial relationships that could be construed as a potential conflict of interest.
