# Supplementary figures and images for: CoQ10 Augments Rosuvastatin Neuroprotective Effect in a Model of Global Ischemia via Inhibition of NF-κB/JNK3/Bax and Activation of Akt/FOXO3A/Bim Cues
Source: Front Pharmacol. 2017 Oct 13;8:735. doi: 10.3389/fphar.2017.00735 (PMC5645536; doi:10.3389/fphar.2017.00735)

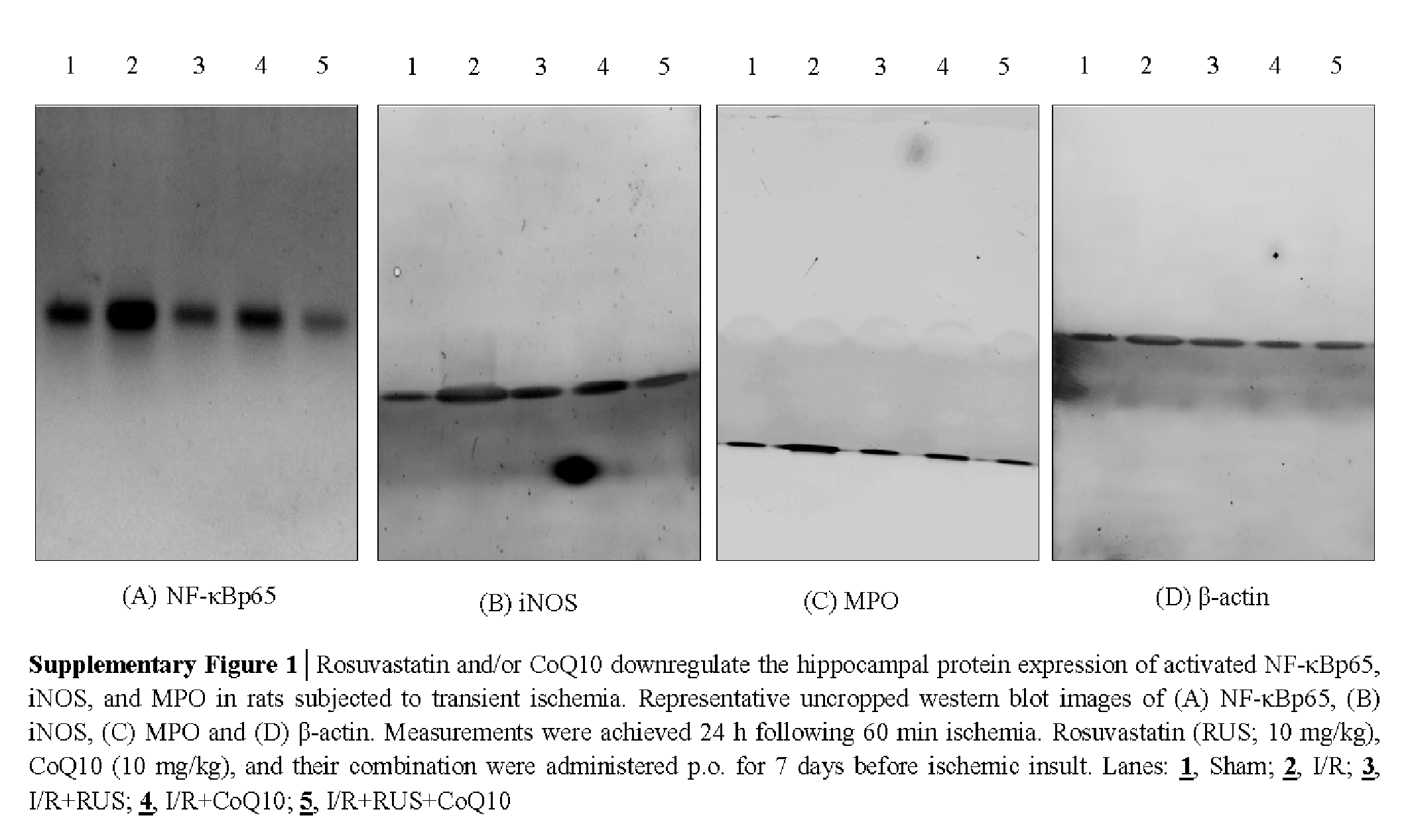

Supplement: Supplementary file 1 [file Image_1.TIF]

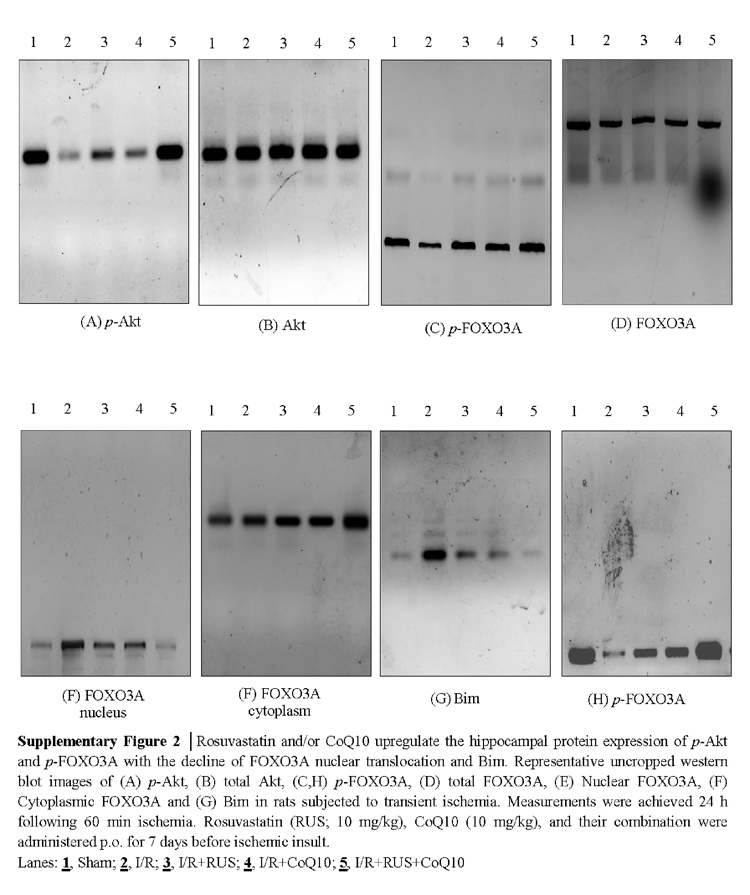

Supplement: Supplementary file 2 [file Image_2.TIF]

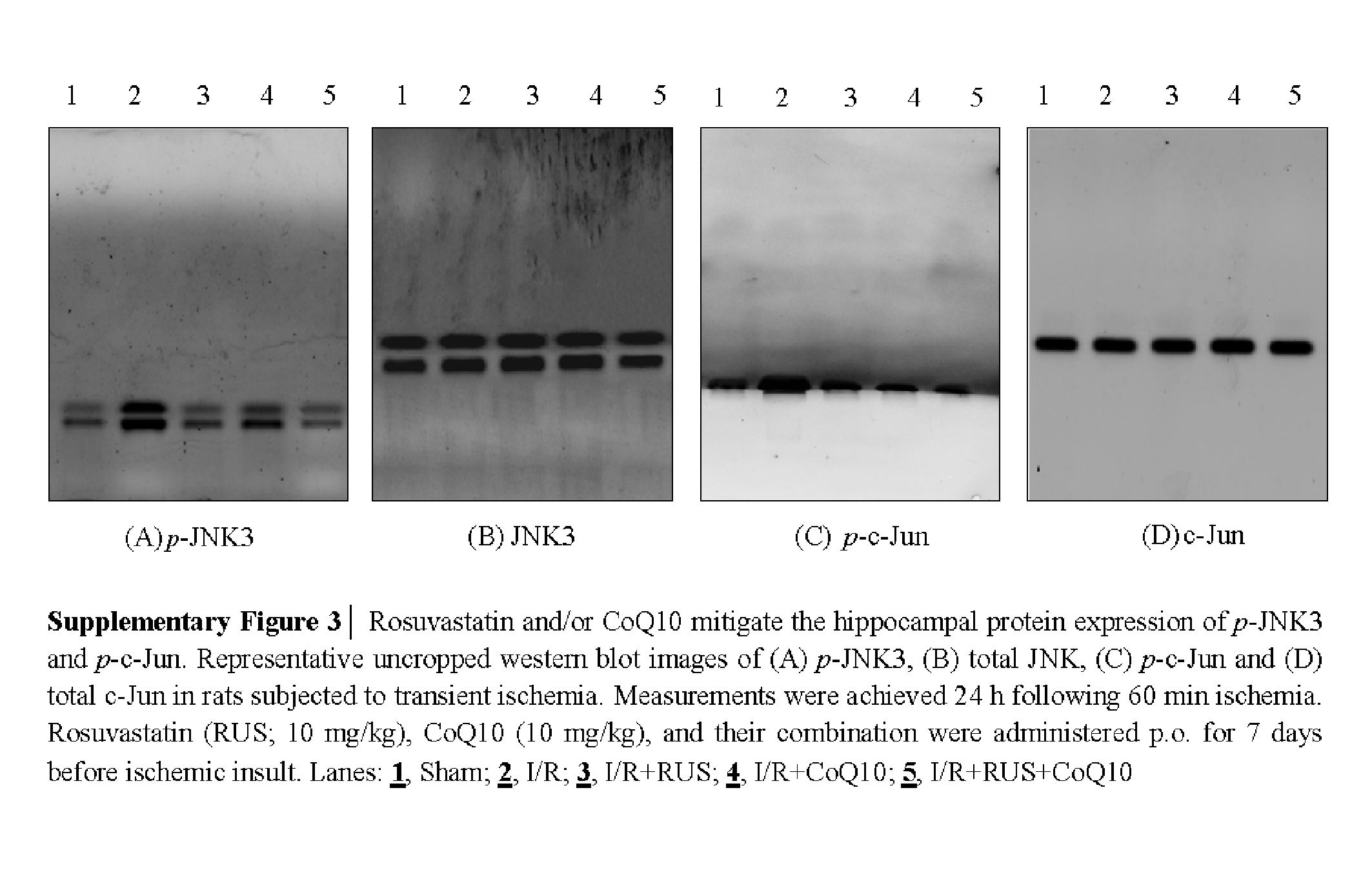

Supplement: Supplementary file 3 [file Image_3.TIF]

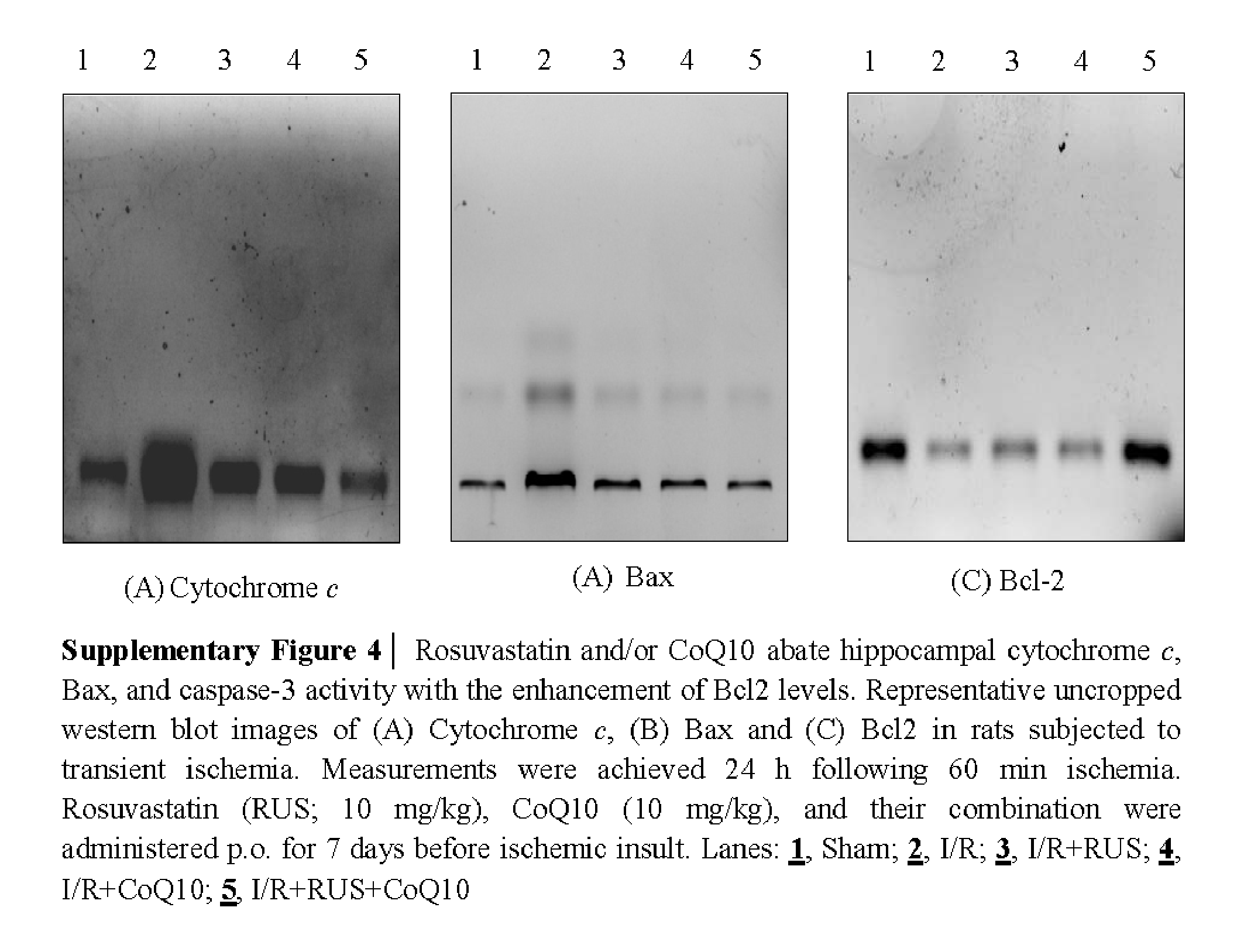

Supplement: Supplementary file 4 [file Image_4.TIF]
